# Transverse Sternal Split: a Safe Mini-invasive Approach for Perventricular Device Closure of Ventricular Septal Defect

**DOI:** 10.21470/1678-9741-2016-0041

**Published:** 2017

**Authors:** Pankaj Garg, Arvind Kumar Bishnoi, Ketav Lakhia, Jigar Surti, Sumbul Siddiqui, Parth Solanki, Himani Pandya

**Affiliations:** 1 Department of Cardiovascular and Thoracic Surgery of the U. N. Mehta Institute of Cardiology and Research Center (affiliated to BJ Medical College, Ahmedabad), Gujarat, India.; 2 Department of Cardiac Anesthesia of the U. N. Mehta Institute of Cardiology and Research Center (affiliated to BJ Medical College, Ahmedabad), Gujarat, India.; 3 Department of Research of the U. N. Mehta Institute of Cardiology and Research Center (affiliated to BJ Medical College, Ahmedabad), Gujarat, India.

**Keywords:** Prosthesis Implantation, Heart Septal Defects, Ventricular, Cardiopulmonary Bypass

## Abstract

**Objective:**

Perventricular device closure of ventricular septal defect through midline sternotomy avoids the cardiopulmonary bypass, however, lacks the cosmetic advantage. Perventricular device closure of ventricular septal defect with transverse split sternotomy was performed to add the cosmetic advantage of mini-invasive technique.

**Methods:**

Thirty-six pediatric patients with mean age 7.14±3.24 months and weight 5.00±0.88 kg were operated for perventricular device closure of ventricular septal defect through transverse split sternotomy in 4^th^ intercostal space under transesophageal echocardiography guidance. In case of failure or complication, surgical closure of ventricular septal defect was performed through the same incision with cervical cannulation of common carotid artery and internal jugular vein for commencement of cardiopulmonary bypass. All the patients were postoperatively followed, and then discharged from hospital due to their surgical outcome, morbidity and mortality.

**Results:**

Procedure was successful in 35 patients. Two patients developed transient heart block. Surgical closure of ventricular septal defect was required in one patient. Mean duration of ventilation was 11.83±3.63 hours. Mean intensive care unit and hospital stay were 1.88±0.74 days and 6.58±1.38 days, respectively. There was no in-hospital mortality. A patient died one day after hospital discharge due to arrhythmia. No patients developed wound related, vascular or neurological complication. In a mean follow-up period of 23.3±18.45 months, all 35 patients were doing well without residual defect with regression of pulmonary artery hypertension as seen on transthoracic echocardiography.

**Conclusion:**

Transverse split sternotomy incision is a safe and effective alternative to a median sternotomy for perventricular device closure of ventricular septal defect with combined advantage of better cosmetic outcomes and avoidance of cardiopulmonary bypass.

**Table:** 

Abbreviations, acronyms & symbols				
ACT	= Activated clotting time		PDA	= Patent ductus arteriosus
CCA	= Common carotid artery		PEM	= Protein energy malnutrition
CHB	= Complete heart block		PTFE	= Polytetrafluorethylene
CPB	= Cardiopulmonary bypass		rSO_2_	= Regional cerebral oxygen saturation
ICU	= Intensive care unit		RA	= Right atrium
IJV	= Internal jugular vein		RV	= Right ventricle
IVC	= Inferior vena cava		SVC	= Superior vena cava
LV	= Left ventricular		TEE	= Transesophageal echocardiography
NIRS	= Near-infrared spectroscopy		TR	= Tricuspid regurgitation
PA	= Pulmonary artery		TTE	= Transthoracic echocardiography
PAH	= Pulmonary artery hypertension		VSD	= Ventricular septal defect

## INTRODUCTION

Surgical closure of ventricular septal defect (VSD) on cardiopulmonary bypass (CPB) in low weight infants is technically challenging and associated with high rate of morbidity and mortality^[1,2]^. Percutaneous device closure is not feasible in these patients, due to technical limitations and high rate of complications. Therefore, hybrid procedure with perventricular device closure is an effective and safe alternative management strategy^[3]^. Hybrid approach is beneficial in avoiding CPB, radiation and complications of vascular access. In addition, there is less need for homologous blood transfusion, 'fast-tracking' - early extubation, mobilization, and hospital discharge^[4,5]^. Perventricular device closure of VSD using transesophageal echocardiography (TEE) guidance on beating euvolemic heart in operating room is a hybrid procedure which offers similar advantages of avoiding ventricular incisions, division of right ventricle (RV) muscle bundles especially moderator band, and immediate confirmation of adequate closure as conventional technique. This technique is safe, particularly in low weight babies, who are high-risk candidates for the procedure in catheterization laboratory^[2,6-8]^.

Perventricular technique, thus, significantly reduces the operative trauma to the patient. However, it is unable to prevent physical and psychological trauma associated with long skin incision and midline sternotomy. Moreover, the use of this technique in case of associated malnutrition, failure to thrive and congestive cardiac failure; a high risk of developing wound infection, mediastinitis, sternal dehiscence, and carinatum deformity can be observed^[4]^.

At our institute, perventricular device closure of VSD using mini-invasive transverse split sternotomy was performed. If patient required surgical closure of VSD, CPB was instituted through cervical cannulation.

## METHODS

This study was carried out between January 2013 to December 2015. Infants with single or predominant, moderate to large muscular or perimembranous VSD with left to right shunt were included in the study. Inclusion criteria's were persistent congestive heart failure despite optimal medical therapy and body weight <10 kg. Patients with any major debilitating illness or additional multiple apical VSDs, who may have required concomitant pulmonary artery banding and patients with associated other cardiac lesions except patent ductus arteriosus (PDA) which required concomitant surgical repair on CPB were excluded. The study was approved by our Institutional Ethics Committee. Parents or guardian were explained in detail about the procedure and informed consent was obtained.

In all the patients, preoperative transthoracic echocardiography (TTE) was performed under sedation to confirm the diagnosis, to assess the size, site and number of VSDs, pulmonary artery hypertension (PAH), ventricular function, suitability of VSD for device closure and to rule out other cardiac lesions.

### Anaesthesia and monitoring

All the infants were operated under general anaesthesia and continuous TEE guidance. Routine invasive hemodynamic monitoring was used in patients requiring VSD closure on CPB, bilateral regional cerebral oxygen saturation (rSO2) was measured using near-infrared spectroscopy (NIRS) (INVOS 5100B, Somanetics, Inc., Troy, MI, USA).

### Perventricular Device Closure of VSD

#### Access and Surgical Procedure (Figure 1)

**Fig. 1:**
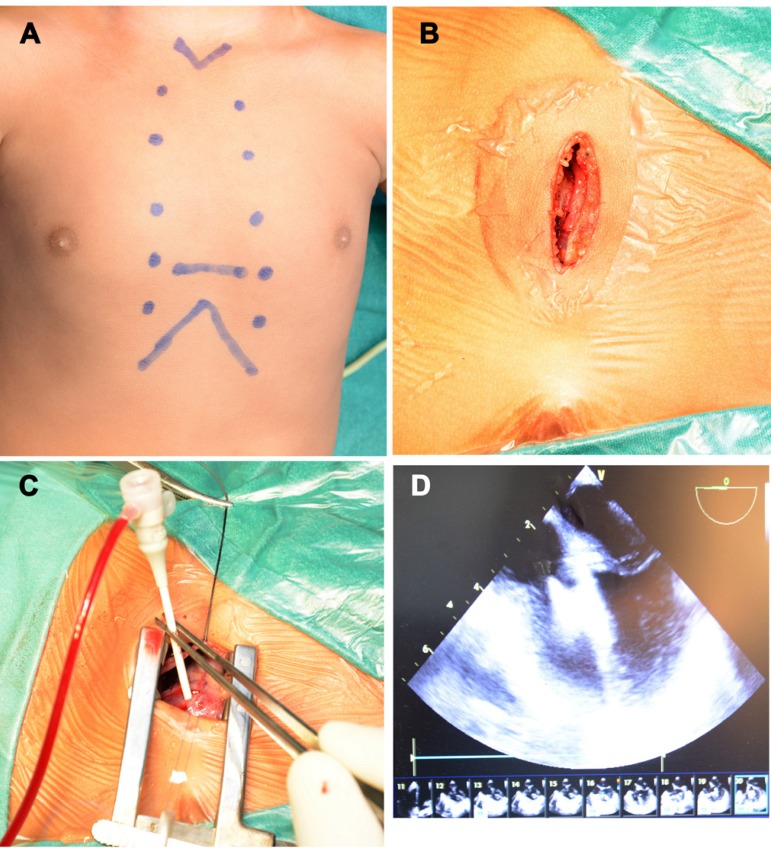
Operative photograph showing proposed incision (A) and transverse sternotomy (B). C - Showing sheath punctured through RV free wall. D - Showing echocardiographic image of VSD device in-situ.

Patients were positioned in supine position with bolster under the scapula, head rest under the head with head turned to the left. Right femoral vein catheterized for central venous access. Draping was done to keep the base of neck, whole of the chest laterally up to posterior axillary line on right side and nipple on the left side exposed (Figure 1A). A transverse skin incision was performed in the 4^th^ intercostal space over the whole width of the sternum and incision deepened to periosteal level. Transverse sternotomy was performed at the same level sparing both internal mammary arteries. Sternotomy was performed at an angle of 45° for better osteo-synthesis and reduced sternal mobility (Figure 1B). Sternal ends were retracted with a small retractor. Thymic gland was divided. Pericardium was opened vertically and pericardial stays were placed.

Under continuous TEE guidance, optimal site for right ventricular puncture was chosen by gentle indentation of RV free wall using finger or forceps-held cotton gauze. Puncture site was considered optimal, if it was away from the papillary muscle and remained at an adequate distance from the septum so as to allow perpendicular access to VSD. A purse string suture was placed on RV free wall at proposed site of puncture with pledgeted 5-0 polypropylene suture. Systemic heparinization was performed with 100 U/kg of unfractionated heparin. A 20G arterial cannula with stylet was introduced into RV, perpendicular to the VSD and stylet was removed. A 0.025-inch straight-tipped guide wire (Terumo Corporation: Tokyo, Japan) was introduced through the cannula and maneuvered across the VSD into the LV (Figure 1C). The cannula was exchanged with gradually enlarging sheath from 5F to finally 8 or 10F (Cordis Corporation; Miami, FL, USA) over 0.038 inch angled guide wire (Cordis Corporation; Miami, FL, USA). Sheath was positioned across VSD with its tip in left ventricular (LV) cavity. Device was advanced into the sheath and deployed under continuous TEE guidance. Imaging in multiple planes was undertaken to confirm appropriate device placement and assess residual shunt (Figure 1D). In addition, any new tricuspid, mitral or aortic valvular obstruction or regurgitation was also looked for. The wires and sheath were removed and the purse string was tied.

#### Selection of VSD Closure Device

Size of VSD was measured by TEE in 4-chamber and long axis views. The greatest of the two values was considered for device selection. VSD occluder device (muscular or perimembranous: mplatz, AGA Medical Corporation, USA; Cardi-O-Fix, Starway Medical Technologies, Inc., Spain) or Duct occluder device (Amplatz, AGA Medical Corporation, Plymouth, MN, USA) 1-2 mm larger than size of VSD was selected. Routinely, muscular VSD device was selected for closure of VSD. Perimembranous VSD device was selected for VSDs in perimembranous location with <5mm muscular margin for device deployment. Duct occluder device was selected for mid-muscular or apical muscular VSDs where it deemed difficult to properly deploy right side of disc due to presence of ventricular trabeculations.

#### Additional Procedure

In patient with associated PDA, anterior approach through the same incision with a metal clip before deployment of VSD device was used for surgical closure.

#### Surgical closure of VSD on CPB Through Cervical Cannulation

A transverse cervical incision 1.5-2 cm in length was performed at one finger's breadth above clavicle between two heads of sternocleidomastoid muscles. Common carotid artery (CCA) and internal jugular vein (IJV) were looped. After heparinisation (400 U/kg), 5 mm polytetrafluorethylene (PTFE) graft was anastomosed to common carotid artery. The graft was cannulated with aortic cannula and connected to arterial line. IJV was clamped cranially and opened transversely. Straight venous cannula was inserted and advanced into the right atrium and connected to venous line.

#### Conduct of CPB (Figure 2)

**Fig. 2:**
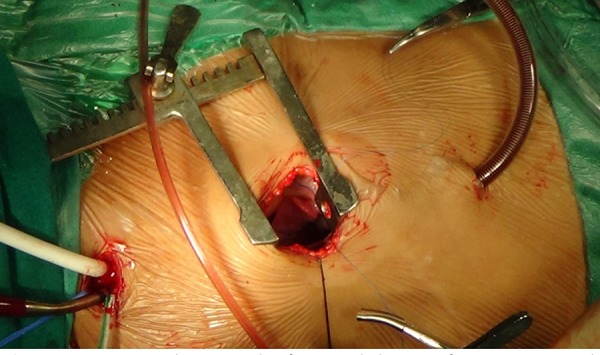
Operative photograph of surgical closure of VSD on CPB with cervical cannulation.

After achieving adequate activated clotting time (ACT), CPB was initiated. Carbon dioxide was continuously insufflated into the operative field throughout the procedure. Inferior vena cava (IVC) was dissected, looped and cannulated with angled venous cannula that was advanced into the pericardial cavity through a stab incision in subxiphoid area. IVC was snugged. Mild hypothermia (30-32°C) was established. De Bakey atraumatic coarctation clamp was applied through a stab incision in the right 2^nd^ intercostal space in mid-axillary line. Del Nido cardioplegia was delivered into the aortic root. IJV cannula was pulled back into superior vena cava (SVC) and SVC was snugged. Right atrium (RA) was opened and a vent was inserted into the left atrium through patent foramen ovale.

#### Surgical Repair

Repair was performed with midline sternotomy approach except that leaflet of tricuspid valve was detached from annulus to expose the borders of VSD. At the end of VSD closure, RA was repaired and SVC and IVC snuggers were removed. Ventricular pacing wire was placed. De-airing was performed and under low flows, aortic cross clamp was removed and IJV cannula was advanced into RA. Time was allowed for the heart to recover the sinus rhythm with good contractility of the ventricles. After re-warming of the patient, IVC cannula was removed and IVC repaired. After confirming adequate de-airing on TEE, cardioplegia puncture site was repaired. Finally, CPB was terminated in usual fashion.

At the end of the procedure, IJV cannula was removed and IJV was repaired with interrupted 6-0 prolene sutures. Heparin was reversed with protamine. PTFE graft was severed near anastomosis with CCA and over sewn to prevent any residual stump. CCA pulsation was confirmed and wound closed.

### Postoperative Care and Follow-Up

All the patients were shifted to intensive care unit (ICU) intubated and managed as per ICU protocol. Postoperative TTE was performed in all the patients before extubation to confirm the adequacy of procedure, residual defect and any complication. Patients were extubated once the clinical condition and arterial blood gases were normal and no evidence of hemolysis or procedural complication on TTE was found. Patients were soon discharged from the ICU and the hospital, if postoperative course was uneventful. All the patients were followed-up with physical examination one week, one month, three months and then six months after surgery to assess the cosmetic result, sternal mobility and any other infirmity. Postoperatively, TTE was performed three months after hospital discharge and at any time during the follow-up period if warranted to evaluate for residual shunt across VSD and pulmonary artery (PA) pressures (estimated by tricuspid regurgitation jet velocity). Residual shunt was classified as trivial (<1 mm), small (1-2 mm), moderate (2-4 mm), or large (>4 mm).

Aspirin (5 mg/kg) was administered for 12 months and oral diuretics (furosemide, 1 mg/kg) for the first three to six months.

### Statistical Analysis

Statistical analysis was carried out using SPSS version 20.0 software (SPSS Inc, USA). The data were presented as mean ± SD, range and in number percentage.

## RESULTS

Table 1 summarizes the demographic profile of the patients.

**Table 1:** Demographic data.

Patients (N)	36
Age (months) (range)	7.14±3.24 (3-14)
Sex (male)	20 (55.5%)
Weight (kg) (range)	5.00±0.88 (2.5-6.3)
Height (cm) (range)	63.41±4.71 (51-71)
BSA (m^2^) (range)	0.29±0.03 (0.18-0.35)
Pre-Saturation (%)	94.94±2.66 (90 -100)
**Preoperative TTE**	
Site of VSD	
Mid-Muscular	23 (63.9%)
Large	15
Moderate	8
Perimembranous	8 (22.2%)
Large	6
Moderate	2
High Muscular (L)	3 (8.3%)
Low Muscular (L)	1 (2.7%)
Posterior Inlet VSD	1 (2.7%)
Additional Defect	14
PDA	5 (35.7%)
Apical VSD	4 (28.6%)
PFO	4 (28.6%)
Preoperative RVSP (mmHg)	83.63±14.73 (50 -112)
Preoperative Severe TR	3 (8.33%)
Ventricular Dysfunction	0

PFO=patent foramen ovale; L=large; M=moderate; BSA=body surface area; TTE=transthoracic echocardiogram; PDA=patent ductus arteriosus; VSD=ventricular septal defect; RVSP=right ventricular septal defect; TR=tricuspid regurgitation

From January 2013 to December 2015, 36 (20 males) symptomatic low weight infants and children with moderate to large muscular or perimembranous VSD on TTE were found suitable for perventricular device closure. Mean age was 7.14±3.24 months and mean weight was 5±0.88 kg. Preoperative saturation on room air was 94.94±2.66%. All patients had persistent clinical signs of congestive cardiac failure despite adequate medical management.

### Preoperative TEE and Location of VSD (Figure 3, Table 1)

**Fig. 3:**
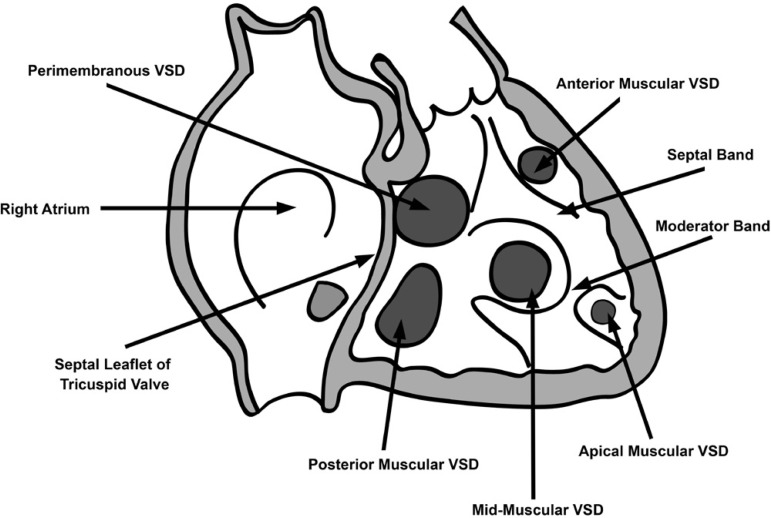
Classification of VSDs: perimembranous and muscular; muscular subdivided into anterior, mid-muscular, posterior and apical. Anterior-anterior to septal band, Mid-muscular-posterior to septal band, Apical-inferior to moderator band, posterior-beneath tricuspid septal leaflet.

Location of VSD was mid-muscular in 23, perimembranous in eight, anterior muscular in three, and posterior muscular in two patients. Mean VSD size was 8.27±1.64 mm in its longest dimension. Additional defects were patent foramen ovale in four patients, small apical muscular VSD in four and PDA in five patients. All except one patient had severe PAH and estimated PA pressures were 83.63±14.73 mmHg. Three patients had severe tricuspid regurgitation (TR). Two patients had mild LV dysfunction.

### Surgical Echocardiographic Outcomes (Table 2)

**Table 2:** Intraoperative, postoperative and follow-up data.

Surgery	36
**Additional Procedure**	
PDA Ligation	5
Duration of Surgery (minutes) (range)	46.66±13.03 (25-85)
Duration of Procedure (minutes) (range)	130.27±25.93 (100-215)
VSD Size (mm) (range)	8.27±1.64 (5-12)
VSD Device Size (mm) (range)	10.91±2.87 (7-16)
**Device**	
Muscular	24 (66.6%)
Perimembranous	9 (25%)
PDA	2 (5.5%)
Guide wire not Passed	1 (2.7%)
**Postoperative Echocardiogram**	
Severe TR	1 (2.7%)
Procedure Success	35 (97.2%)
Surgical Closure	1 (2.7%)
Post RVSP (mmHg) (range)	42.08±12.31 (30-77)
Residual Defect	0
Inotropic Score (range)	3.08±3.38 (0-15)
Ventilation Stay (hrs) (range)	11.83±3.63 (5-20)
ICU Stay (days) (range)	1.88±0.74 (1-3)
Hospital Stay (days) (range)	6.58±1.38 (4-9)
**Postoperative Complications**	
Wound Infection	0
Sternal Dehiscence	0
Temporary Heart Block	2
Permanent Heart Block	0
Mortality	1
Duration of Follow-up (months) (range)	23.30±18.45 (7-42)
**Follow-up Echocardiogram**	0
Residual VSD	0
Severe TR	0

PDA=patent ductus arteriosus; VSD=ventricular septal defect; BSA=body surface area; RVSP=right ventricular septal defect; ICU=intensive care unit; TR=tricuspid regurgitation

Perventricular device closure of VSD was accomplished successfully in 35 patients. Mean procedure time excluding surgical preparation time was 46.66±13.03 minutes. Devices used for VSD closure were muscular in 24 patient, perimembranous in nine patients, and PDA in two patients. Mean VSD occluder device size was 10.91±2.87 mm. Immediately after VSD device closure, 32 patients had no residual shunt; while trivial shunt was detected in three patients. In five patients, additional PDA ligation was accomplished. Estimated mean PA pressure decreased to 42.08±12.31 mmHg (range 30-77 mmHg) on TTE, postoperatively.

### Conversion to CPB

Only one patient of mid muscular VSD required surgical closure, due to inability to cross the guide wire across the VSD. In this patient, patch closure of VSD was accomplished through the same incision and CPB was instituted through cervical cannulation.

### Hospital Length of Stay (Table 2)

**Fig. 4:**
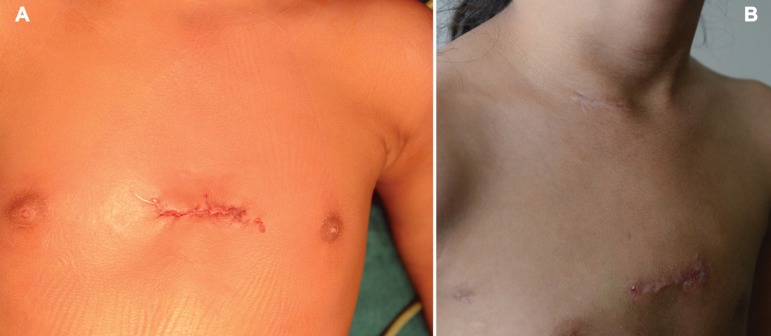
Photograph of final sternal wound closure (A) and healed scar cervical and sternal (B).

Mean duration of ventilation was 11.83±3.63 hours (range 5-20 hours) and mean duration of ICU stay was 1.88±0.74 days (range 1-3 days). Mean inotropic score was 3.08±3.38. Three patients had trivial flow across VSD device. TTE at time of discharge showed severe TR in one patient while LV function was normal in all the patients. All the patients were discharged in stable condition from the hospital and mean duration of hospital stay was 6.58±1.38 days. No patient had any sternal wound related complication (Table 2). Wound closure and final cosmetic result are shown in Figure 4.

### Complications and Deaths

No incidence of device related complications, embolization or LV outflow obstruction was found. No in-hospital death was observed either. A patient died one day after discharge. This patient was operated for device closure of muscular VSD and was discharged in a good condition on the 4^th^ postoperative day. The patient was brought to emergency department by the parents on the following day in a gasping state and died despite resuscitation. The cause of death was unknown as parents refused autopsy, thus, it was presumed to be due to arrhythmias.

### Heart blocks and Arrhythmias

Two patients with perimembranous VSD had prolongation of PR interval following device deployment which recovered spontaneously in few hours. None of the patient developed complete heart block (CHB). However, as a protocol, in all patients, both atrial and ventricular temporary epicardial pacing wires were inserted at the time of closure of sternotomy and left in-situ until the time of discharge.

### Follow-Up

For 35 patients who survived, follow-up was complete. Mean duration of the follow-up period was 23.30±18.45 months. All patients had satisfactory wound healing. Final cosmetic results are shown in Figure 4B. During the follow-up period, all patients were asymptomatic, had weight gain and near complete regression of PA hypertension. Six months after the procedure, all drugs except aspirin were stopped. On repeat echocardiography six months after surgery, none of the patient had shunt across the device and no TR.

## DISCUSSION

Transverse split sternotomy is cosmetically aesthetic, as the incision is small, more obscure, along the Langer's lines and easily hidden especially in females. Also, sternal stability is maintained as sternum is split transversely. It avoids the risk of damage to the mammary gland, pectoral muscle mal-development and scoliosis. Moreover, there is less postoperative pain and bilateral pleural barriers are intact. Thus, it does not compromise spontaneous ventilation. Cervical cannulation for CPB moves almost whole of the hardware away from the operative field. This incision provides equally good exposure of RA, RV, pulmonary arteries and aorta. Therefore, any site of RV free wall puncture can be selected as per the site of VSD. Along with this, PDA can be ligated if required. PA banding can also be easily performed through this approach. However, the need for PA banding as a contraindication for this approach as the patient will require surgical removal of PA band and PA plasty in the future. This will need midline sternotomy and the resulting scar will be cosmetically worse.

For both percutaneous and perventricular device closure of VSD, lower body weight (<5.2 kg) has been found to be the strongest risk factor for procedure failure, device-related complications, and hospital mortality^[9-11]^. Patients in our study were substantially at high risk by virtue of their age, weight, and nutritional status as they were relatively younger (mean age: 7.14±3.24 months), had lower body weight (mean weight: 5±0.88 kg) and poor nutritional status [protein energy malnutrition (PEM) grade III-IV in 85%]. We had 97.2% procedure success with 2.8% incidence of conversion to surgical closure. No in-hospital mortality was observed, however, 30-day mortality was 2.8%. The smallest patient who had successful procedure in our series was 2.5 kg. Our results show that perventricular VSD closure in any location amenable to device can be safely performed using transverse split sternotomy even in high risk patients with acceptable morbidity and mortality. Moreover, in case of procedure failure or any complication; surgical closure of VSD can be performed through the same incision with cervical cannulation without compromising the exposure.

Dreaded complications reported after perventricular device closure of VSD are device embolization^[12-14]^, cardiac perforation, and rarely intra-operative death^[4]^. Other complications are blood loss requiring blood transfusion, hematoma, CHB, ventricular tachyarrhythmia, hypotension, injury of the aortic valve, stroke, and device-related hemolysis^[14-18]^. In our series, we did not encounter any of these complications except two patients who developed transient prolongation of PR interval after device closure of perimembranous VSD that recovered spontaneously. Early or late conduction blocks after device closure are more frequent in patients with <10 kg weight and who have higher device: defect ratio^[19,20]^. We believe that heart blocks can be avoided by careful patient selection, avoiding inlet type of defects and avoiding over sizing the device. Similarly, sustained ventricular arrhythmia or mechanical LV complications can be prevented by gradual up- gradation of sheath size rather than direct insertion of a large sheath into the RV free wall^[2]^. Only one death was observed in our series, an infant who died one day after hospital discharge probably due to ventricular arrhythmia.

Other complications associated with perventricular device closure include LV pseudoaneurysm^[13]^, unexpanded RV disc protruding into pericardium^[5,18]^, incomplete RV disc expansion with screw or disc protruding into the pericardium^[5,21-23]^. None of these complications was found in our series.

## CONCLUSION

Our promising results suggest that perventricular device closure of VSD using mini-invasive transverse split sternotomy in selected high-risk infants is a safe and effective alternative strategy to the conventional perventricular or surgical closure especially in low weight infants.

### Limitation

An observational study with a small number of patients, without any randomized comparison with midline sternotomy arm or surgical closure arm, with a medium-term follow-up period. Patients are still under follow-up for long-term outcomes. This study is, therefore, unable to address incidence of ventricular arrhythmias, ventricular dysfunction, pseudoaneurysm and sudden deaths; complications that have been reported late after the procedure. We recommend randomized control trial with a larger number of patients.

### Lessons Learned

1. Periventricular device closure via transverse split sternotomy is a safe technique even in small and low weight infants.
2. It carries advantages of both conventional and minimal invasive technique.
3. CPB can be established via cervical cannulation in this method and surgical closure of VSD can be performed through the same incision if needed.

**Table:** 

Authors' roles & responsibilities	
PG	Substantial contributions to the conception or design of the work; or the acquisition, analysis, or interpretation of data for the work; agreement to be accountable for all aspects of the work in ensuring that questions related to the accuracy or integrity of any part of the work are appropriately investigated and resolved; final approval of the version to be published
AKB	Substantial contributions to the conception or design of the work; or the acquisition, analysis, or interpretation of data for the work; final approval of the version to be published
KL	Substantial contributions to the conception or design of the work; or the acquisition, analysis, or interpretation of data for the work; final approval of the version to be published
JS	Agreement to be accountable for all aspects of the work in ensuring that questions related to the accuracy or integrity of any part of the work are appropriately investigated and resolved; final approval of the version to be published
SS	Agreement to be accountable for all aspects of the work in ensuring that questions related to the accuracy or integrity of any part of the work are appropriately investigated and resolved; final approval of the version to be published
PS	Agreement to be accountable for all aspects of the work in ensuring that questions related to the accuracy or integrity of any part of the work are appropriately investigated and resolved; final approval of the version to be published
HP	Analysis, or interpretation of data for the work; final approval of the version to be published

## References

[1] Anderson BR, Stevens KN, Nicolson SC, Gruber SB, Spray TL, Wernovsky G (2013). Contemporary outcomes of surgical ventricular septal defect
closure. J Thorac Cardiovasc Surg.

[2] Thakkar B, Patel N, Shah S, Poptani V, Madan T, Shah C (2012). Perventricular device closure of isolated muscular ventricular
septal defect in infants: a single centre experience. Indian Heart J.

[3] Yang XC, Liu DB (2014). Minimally invasive perventricular device closure of ventricular
septal defect: a comparative study in 80 patients. Chin Med Sci J.

[4] Haponiuk I, Chojnicki M, Jaworski R, Steffek M, Juscinski J, Sroka M (2013). Hybrid approach for closure of muscular ventricular septal
defects. Med Sci Monit.

[5] Bacha EA, Cao QL, Galantowicz ME, Cheatham JP, Fleishman CE, Weinstein SW (2005). Multicenter experience with perventricular device closure of
muscular ventricular septal defects. Pediatr Cardiol.

[6] Bacha E, Kalfa D (2014). Minimally invasive paediatric cardiac surgery. Nat Rev Cardiol.

[7] Amin Z, Danford DA, Lof J, Duncan KF, Froemming BS (2004). Intraoperative device closure of perimembranous ventricular
septal defects without cardiopulmonary bypass: preliminary results with the
perventricular technique. J Thorac Cardiovasc Surg.

[8] Yang L, Tai BC, Khin LW, Quek SC (2014). A systematic review on the efficacy and safety of transcatheter
device closure of ventricular septal defects (VSD). J Interv Cardiol.

[9] Kansy A, Tobota Z, Maruszewski P, Maruszewski B (2010). Analysis of 14,843 neonatal congenital heart surgical procedures
in the European Association for Cardiothoracic Surgery Congenital
Database.. Ann Thorac Surg.

[10] Abrishamchian R, Kanhai D, Zwets E, Nie L, Cardarelli M (2006). Low birth weight or diagnosis, which is a higher risk? A
meta-analysis of observational studies. Eur J Cardiothorac Surg.

[11] Holzer R, Balzer D, Cao QL, Lock K, Hijazi ZM, Amplatzer Muscular Ventricular Septal Defect Investigators (2004). Device closure of muscular ventricular septal defects using the
Amplatzer muscular ventricular septal defect occluder: immediate and
mid-term results of a U.S. registry. J Am Coll Cardiol.

[12] Bendaly EA, Hoyer MH, Breinholt JP (2011). Mid-term follow up of perventricular device closure of muscular
ventricular septal defects. Catheter Cardiovasc Interv.

[13] Michel-Behnke I, Ewert P, Koch A, Bertram H, Emmel M, Fischer G (2011). Device closure of ventricular septal defects by hybrid
procedures: a multicenter retrospective study. Catheter Cardiovasc Interv.

[14] Bacha EA, Hijazi ZM (2005). Hybrid procedures in pediatric cardiac surgery. Semin Thorac Cardiovasc Surg Pediatr Card Surg Annu.

[15] Lim DS, Forbes TJ, Rothman A, Lock JE, Landzberg MJ (2007). Transcatheter closure of high-risk muscular ventricular septal
defects with the CardioSEAL occluder: initial report from the CardioSEAL VSD
registry. Catheter Cardiovasc Interv.

[16] Amin Z, Cao QL, Hijazi ZM (2008). Closure of muscular ventricular septal defects: Transcatheter and
hybrid techniques. Catheter Cardiovasc Interv.

[17] Xing Q, Wu Q, Pan S, Ren Y, Wan H (2011). Transthoracic device closure of ventricular septal defects
without cardiopulmonary bypass: experience in infants weighting less than 8
kg. Eur J Cardiothorac Surg.

[18] Holzer R, Marshall A, Kreutzer J, Hirsch R, Chisolm J, Hill S (2010). Hybrid procedures: adverse events and procedural
characteristics--results of a multi-institutional registry. Congenit Heart Dis.

[19] Predescu D, Chaturvedi RR, Friedberg MK, Benson LN, Ozawa A, Lee KJ (2008). Complete heart block associated with device closure of
perimembranous ventricular septal defects. J Thorac Cardiovasc Surg.

[20] Diab KA, Cao QL, Mora BN, Hijazi Ziyad M (2007). Device closure of muscular ventricular septal defects in infants
less than one year of age using the Amplatzer devices: feasibility and
outcome. Catheter Cardiovasc Interv.

[21] Bacha EA, Cao QL, Starr JP, Waight D, Ebeid MR, Hijazi ZM (2003). Perventricular device closure of muscular ventricular septal
defects on the beating heart: technique and results. J Thorac Cardiovasc Surg.

[22] Crossland DS, Wilkinson JL, Cochrane AD, d'Udekem Y, Brizard CP, Lane GK (2008). Initial results of primary device closure of large muscular
ventricular septal defects in early infancy using perventricular
access. Catheter Cardiovasc Interv.

[23] Gan C, Lin K, An Q, Tang H, Song H, Lui RC (2009). Perventricular device closure of muscular ventricular septal
defects on beating hearts: initial experience in eight
children. J Thorac Cardiovasc Surg.

